# Health Personality, Physical and Emotional Well-Being

**DOI:** 10.1093/geroni/igaa057.2166

**Published:** 2020-12-16

**Authors:** Rotem Arieli

**Affiliations:** Iowa State University, Ames, Iowa, United States

## Abstract

The purpose of this study was to identify paths from health personality to outcomes of physical and emotional health. Data included 3,907 participants, 65 and older. Latent path models conducted in Mplus resulted in several significant pathways. Health neuroticism, health extraversion, and health openness negatively predicted both outcomes of physical and emotional health significantly. This negative association indicates an inverse relationship, meaning the more worried older adults were about their health, the lower their self-rating of physical and emotional health. Health agreeableness was negatively predictive of physical health, but not emotional health. Health conscientiousness had a significant positive association with both physical and emotional health, indicating that the more conscientious participants were about their health, the better their physical and emotional health. This study’s findings can be translated to targeted intervention programs for emotional and physical health outcomes benefiting older adults.

